# Beneficial Metabolic Effects of 2′,3′,5′-tri-acetyl-N_6_- (3-Hydroxylaniline) Adenosine in the Liver and Plasma of Hyperlipidemic Hamsters

**DOI:** 10.1371/journal.pone.0032115

**Published:** 2012-03-28

**Authors:** Yang Sun, Zeqin Lian, Chunying Jiang, Yinghong Wang, Haibo Zhu

**Affiliations:** State Key Laboratory of Bioactive Substances and Functions of Natural Medicines, Institute of Materia Medica, Chinese Academy of Medical Sciences and Peking Union Medical College, Beijing, China; The George Washington University, United States of America

## Abstract

**Background:**

Pharmaceutical research of hyperlipidemia has been commonly pursued using traditional approaches. However, unbiased metabonomics attempts to explore the metabolic signature of hyperlipidemia in a high-throughput manner to understand pathophysiology of the disease process.

**Methodology/Principal Findings:**

As a new way, we performed ^1^H NMR-based metabonomics to evaluate the beneficial effects of 2′,3′,5′-tri-acetyl-N_6_- (3-hydroxylaniline) adenosine (WS070117) on plasma and liver from hyperlipidemic Syrian golden hamsters. Both plasma and liver profiles provided a clearer distinction between the control and hyperlipidemic hamsters. Compared to control animals, hyperlipidemic hamsters showed a higher content of lipids (triglyceride and cholesterol), lactate and alanine together with a lower content of choline-containing compounds (e.g., phosphocholine, phosphatidylcholine, and glycerophosphocholine) and betaine. As a result, metabonomics-based findings such as the PCA and OPLS-DA plotting of metabolic state and analysis of potential biomarkers in plasma and liver correlated well to the assessment of biochemical assays, Oil Red O staining and *in vivo* ultrasonographic imaging suggesting that WS070117 was able to regulate lipid content and displayed more beneficial effects on plasma and liver than simvastatin.

**Conclusions/Significance:**

This work demonstrates the promise of applying ^1^H NMR metabonomics to evaluate the beneficial effects of WS070117 which may be a good drug candidate for hyperlipidemia.

## Introduction

A novel adenosine analog, 2′,3′,5′-tri-acetyl-N6-(3-hydroxylaniline) adenosine (WS070117) displays anti-hyperlipidemic activity in preliminary *in vivo* and *in vitro* experiments [1]. Serum lipids contents, such as TC, TG, LDL-C, significantly reduced after WS070117 treatment in high fat diet fed hamster. But these biochemical evidences is not sufficient to figure out the difference of metabolic profile of WS070117 when compared with other lipid lowing agents.

Metabonomics is a global metabolite profiling approach for biological samples, particularly, biofluids. It can be readily applied to monitor the changes in metabolite concentration and profiles in response to physiological and non-physiologic challenges such as drugs or toxins [2]. Applications of metabonomic technologies are increasing exponentially in biological studies such as physiological genomics and potential lead target validation [3]–[5], mechanistic toxicology [6], [7], clinical pharmacology [8], and disease investigation [9]–[11].

Nuclear magnetic resonance spectroscopy (NMR)-based metabonomics is non-discrimiating, non-invasive and little sample preparation required which can reveal structural information for identification of an unknown metabolite which may be a new biomarker. Previous studies have examined the ability of NMR-based metabonomic analysis of plasma and liver constituents to investigate the metabolic profiling of different model animals with hyperlipidemia related disease [12], [13].

Here, we evaluated the applicability of NMR-based metabonomics in assessing the effects of WS070117 on hyperlipidemic hamsters conformed by other traditional methods as well as sought to identify the potential biomarkers helpful to dissect the underlying efficacies and mechanisms of WS070117. Our results showed that WS070117 induced changes in lipids, lipid metabolism-related molecules, glucose, and some key amino acids in plasma and liver in hyperlipidemic hamsters, suggesting that this novel compound may be a good drug candidate for hyperlipidemia.

## Results

### Serum and hepatic biochemical parameters

**Figure 1:**
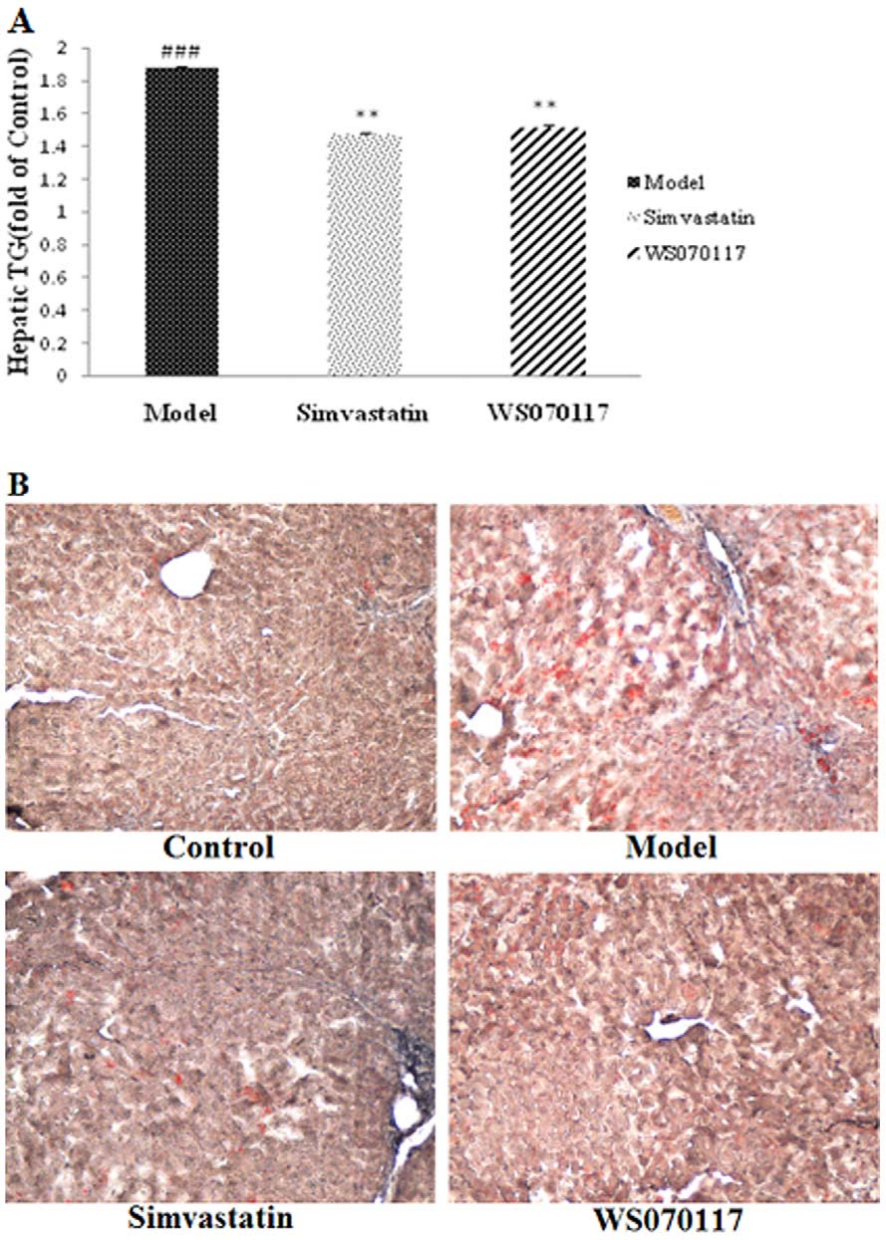
WS070117 prevented liver fat accumulation in hyperlipidemic hamsters. (A): The effect of WS070117 on hepatic TG at week 10 (n = 5). (B): WS070117 reduced lipid droplets in the liver of hyperlipidemic hamsters. ## *P*<0.001, compared with control group, **P<0.01, compared with model group.

The hamster, which has been established for studying diet-induced hyperlipidemia [14], [15], is considered to be a suitable model for humans to study the hyperlipidemia induced by a high-fat diet (HFD), since human and Syrian golden hamsters lipoprotein and apolipoprotein profiles are similar when hamsters are fed a high-fat diet. Herein, hamsters fed a high fat diet for 2 weeks were used to develop a dyslipidemia model (Table 1). The liver/body weight index was markedly increased for the HFD-fed animals at the end of the experiment. However, the indexes of the simvastatin and WS070117 treated groups were significantly lower. HFD also caused a marked increase in serum total cholesterol (TC), triglyceride (TG) and low density lipoprotein cholesterol (LDL-C) concentrations (Table 1). WS070117 treatment resulted in a significant decrease in serum TC, TG, and LDL-C concentrations. Hepatic TG concentrations were also reduced by WS070117 (Fig. 1A). Moreover, the efficacy of WS070117 was comparable to that of the widely used hypolipidemic agent simvastatin when treated with the same dose.

**Table 1:** Lipid-lowering effects of WS070117 (2 mg·kg^−1^) in serum from hyperlipideminc hamsters.

Period	Parameters	Control	Model	Simvastatin	WS070117
**Model** **Construction**	**TG (mmol·L^−1^)**	2.11±0.46	3.90±1.11###	4.05±0.61###	3.57±0.67###
	**TC (mmol·L^−1^)**	2.65±0.16	5.59±1.05###	5.70±0.72###	5.70±0.65###
	**TG (mmol·L^−1^)**	1.85±0.30	9.76±1.50###	7.86±0.54*	4.30±0.70***
	**TC (mmol·L^−1^)**	2.86±0.21	7.73±1.08###	5.87±0.47**	4.51±0.37***
**Drug** **Treatment**	**LDL-C(mmol·L^−1^)**	0.92±0.15	3.23±0.44###	2.33±0.28**	1.44±0.30***
	**HDL-C(mmol·L^−1^)**	1.06±0.19	2.12±0.47^##^	1.64±0.30	1.68±0.27
	**Body weight**	136±14.2	182±18.7###	181±23.7	192±16.9
	**Liver/BW**	0.039±0.0055	0.048±0.0034###	0.046±0.0018	0.046±0.0056

The mean ± SD of 5 animals are presented.

### *P*<0.001, compared with control;

* *P*<0.05,

** *P*<0.01,

*** *P*<0.001, compared with model.

### Ultrasonographic imaging

Ultrasonographic imaging is a non-invasive, inexpensive, and repeatable technique used to detect fat accumulation in the liver. Although liver ultrasonographic imaging does not provide accurate quantification of the amount of fat present and does not allow the severity of histological disease to be established, it is still a non-invasive and repeatable technique to detect changes in liver parenchyma. Normal liver has a homogeneous distribution and the echogenicity of the entire structure is similar. Liver parenchyma is characterized by areas with different echogenicity [16]. Fat accumulation in liver causes increased echogenicity, and thus the liver appears smooth and brighter. Furthermore, echo shadows tend to be coarser in the presence of diffuse hepatic steatosis. Differences in image texture are an important indicator in the detection of a fatty liver.

**Figure 2:**
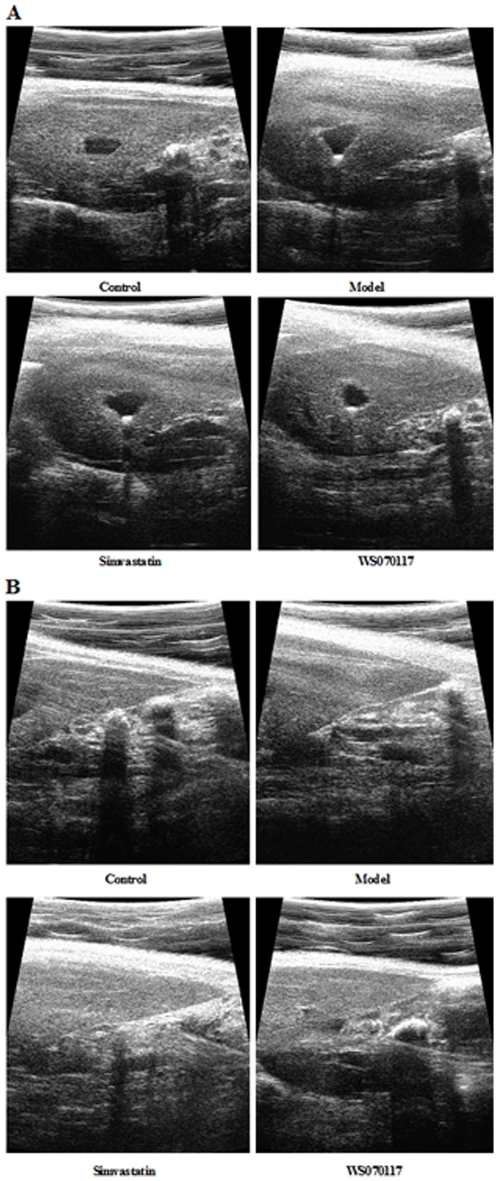
Various echogenicity in hamster liver tissue demonstrates that WS070117 prevents fat accumulation in the liver. (A) Images of pericholecystic space; (B) Images of liver edge.

The assessment of irregular and nodular surfaces, blunt edges, and parenchymal abnormalities was independently blinded reviewed and confirmed by three experienced experts according to the unified impression for the whole imaging manifestations. The obviously morphological changes of steatosis include gallbladder blurring and blunt liver edge in HFD hamsters. By ultrasonographic assay, compared to that of the model animals, the livers from hyperlipidemic hamsters treated with simvastatin or WS070117 had attenuated echogenicity, suggesting that treatment reduced fat accumulation (Fig. 2).

### Oil Red O staining estimation of liver fat accumulation

Oil Red O staining was performed to estimate hepatic fat content. The hepatocytes of animals in the HFD-fed hyperlipidemic hamsters were compressed and separated by bulks of fat, and the livers showed a strikingly pale yellow color, indicating an abnormally high level of fat accumulation and deposition. Treatment with simvastatin or WS070117 substantially repressed these changes, and the livers from hyperlipidemic hamsters treated with simvastatin or WS070117 had a dark red appearance similar to that of the control animals (Fig. 1B).

### Metabolite identification

With the results above, we evaluated the beneficial effects of WS070117 by metabonomics using plasma and liver samples. NMR-based metabonomics is a global metabolite profiling approach for further systemic analysis. Moreover, NMR spectroscopy can give structural information about the potential biomarkers. Using ^1^H NMR spectroscopy, we investigated the metabolic profiles of liver and plasma samples and a list of identified metabolites are shown in Table S1.

**Figure 3:**
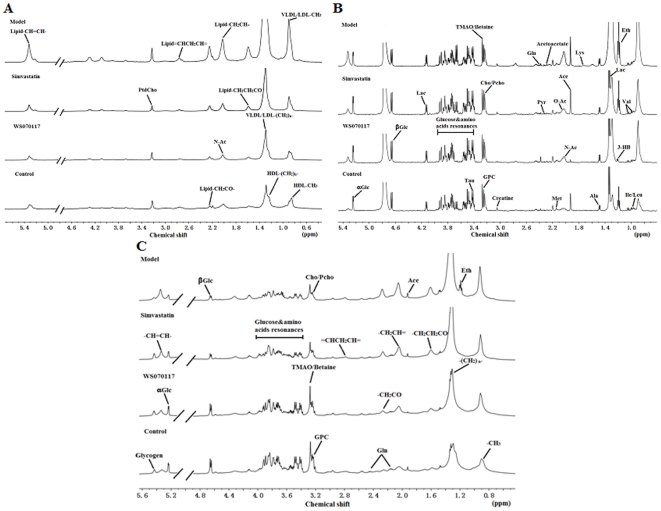
Typical 500 MHz ^1^H-NMR spectra of hamster plasma and liver samples. (A): ^1^H-NMR BPP-LED spectra of plasma samples. (B): ^1^H-NMR CPMG spectra of plasma samples. (C): HR-MAS ^1^H-NMR standard spectra of liver tissues. Signal assignment: VLDL, very low-density lipoprotein; LDL, low-density lipoprotein; HDL, high-density lipoprotein; PtdCho, Phosphatidylcholine; Ile, Isoleucine; Leu, Leucine; 3-HB, 3-D-Hydroxybutyrate; Val, Valine; Eth, (residual) Ethanol; Lac, Lactate; Ala, Alanine; Lys, Lysine; Ace, Acetate; N-Ac, N-Acetyl glycoproteins; O-Ac, O-Acetyl glycoproteins; Met, Methionine; Pyr, Pyruvate; Gln, Glutamine; Cho, Choline; GPC, Glycerophos- phorylcholine; Glc, Glucose; TMAO, Trimethylamine-N-oxide.

Although there were some apparent differences between these representative spectra based on simple visual inspection (Fig. 3), they were not consistent across all samples. Therefore, we applied a multivariate statistical method to analyze the spectra in a more holistic way and to identify signals that can efficiently differentiate the groups.

### Multivariate analysis for the potential biomarkers

#### Metabolic changes in the plasma

After being processed by Principal Components Analysis (PCA) and Partial Least Squares-Discriminant Analysis (PLS-DA) in the software SIMCA-P package, mean-centered PCA and PLS-DA score plots could be generated to trace and compare the dynamic recovery of metabolic events in hamsters. In the PCA and PLS-DA maps, each spot represented a sample and each assembly of samples indicated a particular metabolic pattern in different state.

**Figure 4:**
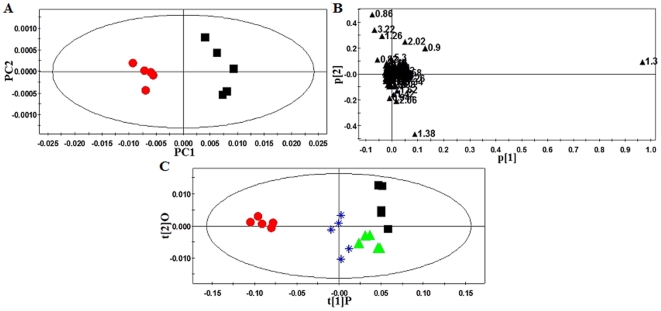
PR analysis of ^1^H-NMR BPP-LED spectra of hamster plasma. (A): PCA scores plot of ^1^H-NMR BPP-LED spectra of plasma samples from normal and hyperlipidemic hamsters (R^2^X = 0.998, Q^2^ = 0.808). (B): Corresponding loadings plot indicating those NMR spectral regions responsible for the separation. (C): Scores plot of OPLS-DA analysis of the spectra of hamster plasma (R^2^X = 0.994, R^2^Y = 0.518, Q^2^ = 0.347). •, Control hamsters; ▪, Hyperlipidemic hamsters; ▴, Hyperlipidemic hamsters treated with simvastatin (2 mg·kg^−1^); *, Hyperlipidemic hamsters treated with WS070117 (2 mg·kg^−1^).

The PCA scores and loadings plot of the ^1^H Bipolar pulse pair-longitudinal eddy current delay (BPP-LED) NMR data of plasma samples showed an apparent higher level of low density lipoprotein/very low density lipoprotein (LDL/VLDL) (δ0.9,1.30 ppm), O-acetyl glycoproteins (O-Ac, δ2.06 ppm) and some of the lipid signals, including CH_2_-CH_2_CO (δ1.58 ppm), CH_2_-CH = (δ2.02 ppm), CH = CH (δ5.34 ppm), and CH_2_-CO (δ2.26 ppm), accompanied with a lower level of high density lipoprotein (HDL) (δ0.86,1.26 ppm) and phosphatidylcholine (PtdCho) (δ3.22 ppm) in the plasma samples of hyperlipidemic hamsters (Fig. 4A, B and Table 2).

**Table 2:** Relative integrals from some selected metabolites contributing to the classification of hamsters in four groups.

Biological matrices	Metabolites	Control	Model	WS070117	Simvastatin	p- value
Plasma	HDL&	0.046±0.0022	0.030±0.0024	0.037±0.0057	0.034±0.0054	4.7×10^−6a^, 0.036^b^, 0.17^c^
	LDL/VLDL&	0.035±0.0027	0.059±0.0037	0.051±0.0050	0.058±0.0060	2.4×10^−6a^, 0.019^b^, 0.92^c^
	Lactate&	0.038±0.0042	0.10±0.0034	0.075±0.0063	0.090±0.0089	3.9×10^−9a^, 2.5×10^−5b^, 0.013^c^
	Alanine&	0.0013±0.00044	0.0034±0.0006	0.0022±0.0004	0.0031±0.0005	2.0×10^−4a^, 0.0063^b^, 0.34^c^
	O-Acetyl glycoproteins&	0.016±0.0010	0.020±0.0007	0.017±0.0012	0.018±0.0011	1.3×10^−4a^, 0.0039^b^, 0.040^c^
	Betaine&	0.0049±0.0009	0.0027±0.0003	0.0048±0.0013	0.0038±0.0011	6.2×10^−4a^, 0.0084^b^, 0.083^c^
	PtdCho&	0.023±0.0020	0.010±0.0009	0.018±0.0039	0.014±0.0040	1.0×10^−6a^, 0.0019^b^, 0.055^c^
	GPC&	0.031±0.0042	0.018±0.0036	0.028±0.0044	0.022±0.0089	9.4×10^−4a^, 0.0046^b^, 0.39^c^
Liver	Lipids-CH_3_ &	0.018±0.0018	0.026±0.0040	0.024±0.0033	0.026±0.0039	0.0032^a^, 0.49^b^, 0.96^c^
	Lipids-(CH_2_)_n_ &	0.031±0.0048	0.068±0.0131	0.055±0.0161	0.063±0.0131	3.3×10^−4a^, 0.32^b^, 0.73^c^
	Lipids-CH_2_CH = &	0.0092±0.00082	0.012±0.0014	0.011±0.0013	0.012±0.0013	0.0048^a^, 0.35^b^, 0.77^c^
	Lactate&	0.030±0.0022	0.073±0.0173	0.056±0.0178	0.063±0.0065	6.2×10^−4a^, 0.19^b^, 0.35^c^
	Alanine&	0.013±0.0015	0.017±0.0020	0.014±0.0006	0.017±0.0015	0.013^a^, 7.1×10^−4b^, 0.47^c^
	Betaine&	0.022±0.0032	0.016±0.0022	0.020±0.0013	0.018±0.0012	0.010^a^, 0.0052^b^, 0.080^c^
	GPC&	0.011±0.0021	0.0079±0.0014	0.0092±0.0012	0.0083±0.0005	0.015^a^, 0.086^b^, 0.38^c^

& , normalized to the total of all the resonance integral regions over the range of 0.5–6.0 ppm excluding the resonance from residual water (4.7–5.1 ppm);

a , Model group compared with Control;

b , WS070117 (2 mg·kg^−1^) treated group compared with Model;

c , Simvastatin (2 mg·kg^−1^) treated group compared with Model.

We further analyzed the data with the Orthogonal Partial Least Squares-Discriminant Analysis (OPLS-DA) multivariate approach, which can separate groups in the presence of large structured noise [17], [18]. The model had an overall goodness of fit, R^2^(Y), of 97% and an overall cross-validation coefficient, Q^2^(Y), of 76%. All hyperlipidemic hamsters exhibited distinct plasma metabolic profiles after a 10-week course of treatment with simvastatin or WS070117. There was a remarkable decrease in lipid signals (e.g., LDL/VLDL, etc), suggesting that both simvastatin and WS070117 reversed the plasma metabolic changes caused by hyperlipidemia. Importantly, only WS070117 treatment made a significantly statistical difference compared with simvastatin, suggesting WS070117 had more pronounced hypolipidemic effects (Fig. 4C, Table 2).

PCA analysis identified metabolic changes in the ^1^H Carr-Purcell-Meiboom-Gill (CPMG)-NMR spectra of plasma samples of control and hyperlipidemic hamsters; these included an increase in the signal intensities of lactate (δ1.34 ppm), alanine (δ1.46 ppm) and some lipids such as LDL/VLDL (δ0.9, 1.30 ppm) and CH = CH (δ5.34 ppm), accompanied by a signal reduction of betaine (δ3.26 ppm) and glycerophosphocholine (GPC) (δ3.22 ppm) in hyperlipidemic animals.

In order to select potential biomarkers worthy of preferential study, these differential metabolites were validated using Student's t test. The critical p-value was set to 0.05 for significantly differential variables in this study. The alterations of peak intensity of the selected potential biomarkers of three groups were summarized in Table 2. It was obvious that most of the biomarkers in WS070117 group revealed a higher degree of recovery after 10 weeks' therapeutic intervention.

#### Metabolic changes in intact liver

**Figure 5:**
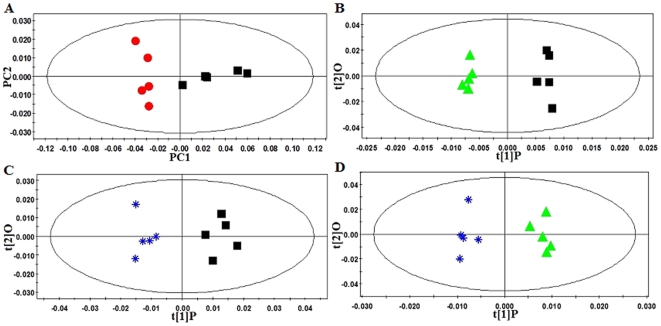
PR analysis of HR-MAS ^1^H-NMR standard spectra of hamster liver tissues. (A): PCA analysis of the spectra of liver tissues from normal and hyperlipidemic hamsters (R^2^X = 0.997, Q^2^ = 0. 945). (B), (C) and (D): Scores plot of pair-wised OPLS-DA analysis of the spectra of liver tissues from untreated and treated hyperlipidemic hamsters ((B) R^2^X = 0.98, R^2^Y = 0.97, Q^2^ = 0.762; (C) R^2^X = 0.979, R^2^Y = 0.942, Q^2^ = 0.723; (D) R^2^X = 0.988, R^2^Y = 0.968, Q^2^ = 0.838). •, Control hamsters; ▪, Hyperlipidemic hamsters; ▴, Hyperlipidemic hamsters treated with simvastatin (2 mg·kg^−1^); *, Hyperlipidemic hamsters treated with WS070117 (2 mg·kg^−1^).

The liver metabolic profiles of hyperlipidemic and control hamsters obtained by magic-angle spinning nuclear magnetic resonance (HR-MAS) ^1^H-NMR spectra displayed clear differences (Fig. 5A). PLS-DA score plot was characterized by R^2^ = 0.99 and Q^2^ = 0.95. This higher Q^2^ value indicated good predictive capabilities of the model. The predominant changes identified in the PLS-DA analysis of livers from hyperlipidemic animals included an increase in the signal intensities of lactate (1.34 ppm), alanine (1.46 ppm) and some lipid signals such as -CH_3_, -(CH_2_)_n_-,-CH_2_CH = (δ0.90, 0.94, 1.30, 1.34 and 2.02 ppm), accompanied by a reduction in the intensity of betaine (δ3.26 ppm) and choline-containing compounds (e.g., phosphocholine, GPC) (δ3.22 ppm) (Fig. 5A). Treating hyperlipidemic hamsters with simvastatin or WS070117 reverts some of the hyperlipdemic effects on the metabolite concentrations (Fig. 5B and C).

OPLS-DA of scores plot representing hamsters treated with simvastatin or WS070117 shifted away from each other, indicating that different metabolic patterns have been established due to distinct interference, and the corresponding loadings plots indicated that WS070117 reduced lipid accumulation in the liver (Fig. 5D), which agrees with the Oil Red O staining results (Fig. 1B).

The changes of peak intensity of the selected potential biomarkers of three groups were also listed in Table 2. It revealed that most of the biomarkers in WS070117 group revealed a higher degree of recovery after 10 weeks' administration.

#### Metabolic changes in the liver extracts

**Figure 6:**
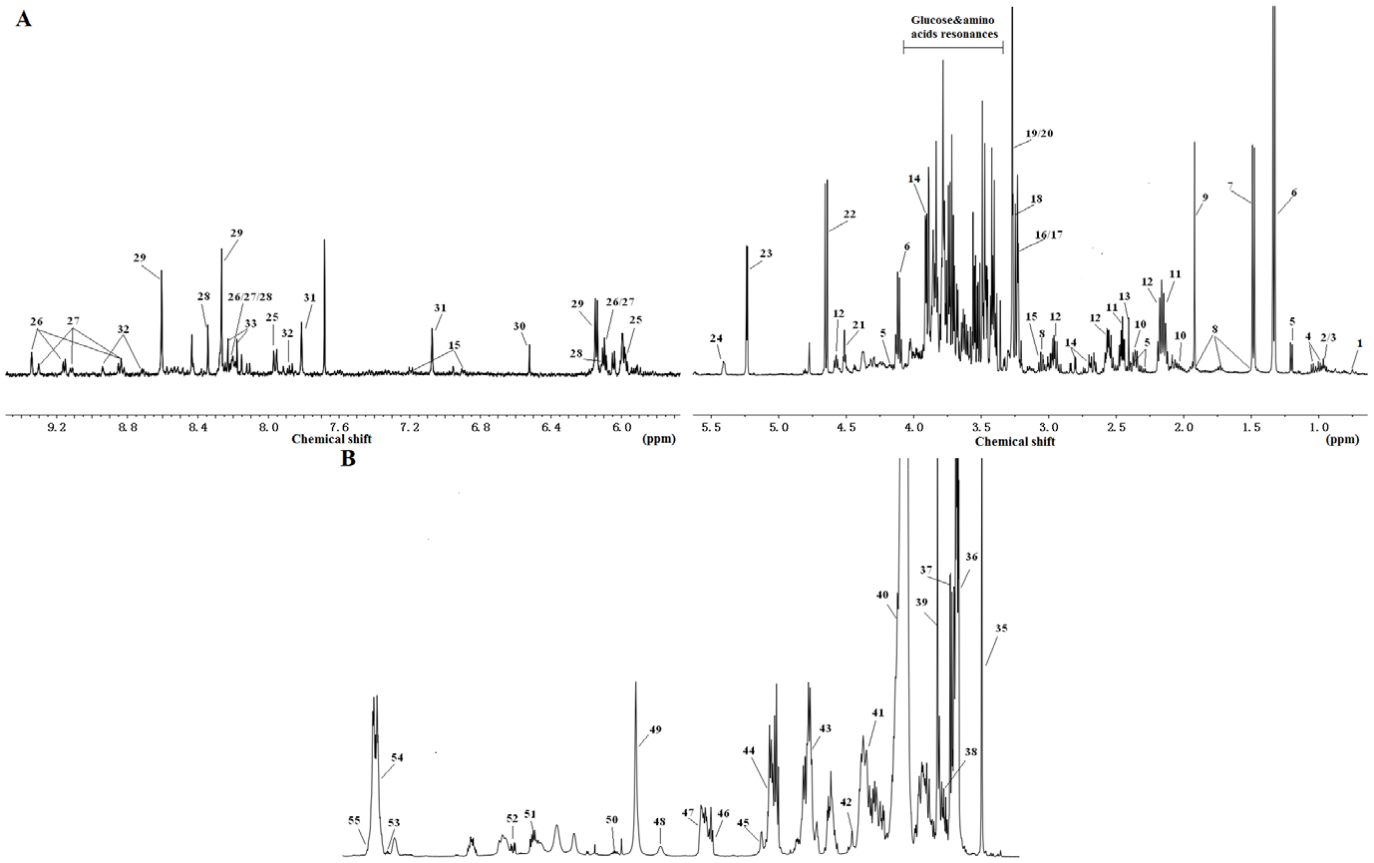
High resolution 500 MHz single pulse ^1^H-NMR spectra of liver tissue extracts from control hamsters. (A): ^1^H-NMR spectrum of liver tissue aqueous extracts from control hamsters. (B): ^1^H-NMR spectrum of liver tissue chloroform/methanol extracts from control hamsters. Signal assignment: 1, Bile acid C18 methyls; 2, Isoleucine; 3, Leucine; 4, Valine; 5, β-Hydroxybutyrate; 6, Lactate; 7, Alanine; 8, Lysine; 9, Acetate; 10, Glutamate; 11, Glutamine; 12, Glutathione; 13, Succinate; 14, Aspartate; 15, Tyrosine; 16, Choline; 17, Phosphocholine; 18, Glycerophosphorcholine; 19,Trimethylamine-N-oxide; 20, Betaine; 21, N-methyl nicotinamide; 22, β-Glucose; 23, α-Glucose; 24, Glycogen; 25, UDP-Glucose; 26, NAD^+^; 27, NADP^+^; 28, Inosine&adenosine; 29, AMP; 30, Fumarate; 31, Histidine; 32, Nicotinurate; 33, Adenine; 34, Adenosine; 35, Cholesterol (C_18_H_3_); 36, Fatty acid residues (ω-CH_3_); 37, Cholesterol (C_26_H_3_, C_27_H_3_, C_21_H_3_); 38, Fatty acid residues (ω-CH_3_ of total omega-3 fatty acid); 39, Cholesterol (C_19_H_3_); 40, Fatty acid residues ((CH_2_)_n_); 41, Fatty acid residues (COCH_2_-CH_2_); 42, Fatty acid residues (-CH_2_ of ARA+EPA); 43, Fatty acid residues (CH2-CH = ); 44, Fatty acid residues(-CO-CH_2_); 45, Fatty acid residues (α and β CH_2_ of DHA); 46, Fatty acid residues (-CH = CH-CH2-CH = CH-of linoleic acid); 47, Fatty acid residues (CH = CH-CH2-CH = CH)_n_, n>1; 48, CH_2_-NH_2_ of PE; 49, N^+^(CH_3_)_3_ (PC and SM); 50, Cholesterol (C_3_H); 51, Triglycerides (C_1_H and C_3_H of glycerol); 52, Triglycerides (C_1_H and C_3_H of glycerol); 53, Triglycerides (C_2_H of glycerol); 54, Fatty acid residues (CH = CH); 55, Cholesterol (C_6_H). ARA, Arachidonic acid; EPA, Eicosapentaenoic acid; DHA, Doco- sahexaenoic acid; PE, Phosphatidylethanolamine; PC, Phosphatidylcholine; SM, Sphingo- myelin.

To further study the differences between the lipid profiles of each hamster group, the spectra of liver aqueous and lipophilic extracts were obtained (Fig. 6). Metabolic profiles of the hydrophilic extracts of hyperlipidemic hamsters and control animals were mostly the same, as assessed by the ^1^H CPMG-NMR spectra. Signals attributed to cholesterol (in free and esterified form) triglycerides, phospholipids and fatty acid residues comprised the total lipid extract NMR fingerprint (Fig. 6B). The main differences between hyperlipidemic and control hamsters were related to the structural features of the fatty acids and cholesterol.

The HR-MAS ^1^H-NMR spectrum revealed information on metabolites found in both aqueous and lipophilic extracts; detailed analysis suggested a pronounced difference in the content of hepatic lipids and choline-containing compounds between WS070117 treated and untreated hyperlipidemic golden hamsters. Not surprisingly, the metabolic differences between simvastantin and WS070117 treated hyperlipidemic hamsters were consistent with the results observed in intact livers by HR-MAS NMR (Fig. 5).

## Discussion

Based on the results of conventional pharmacological methods, it was found that the hyperlipidemic hamster model induced by HFD had been successfully established. Treatment with WS070117 made a significant reverse that resulted in decreases serum TC, TG, LDL-C and hepatic TC and TG. The liver Oil Red O staining and ultrasonographic image data show that WS070117 significant decrease the liver lipid accumulation in hyperlipidemic hamsters. These evidences were concluded that WS070117 had a lipid-regulating effect on both plasma and liver. Simvastatin perform the similar lipid lowing effect in this model. However, the above results were difficult to figure out the advantage of WS070117 compared with simvastatin at the same dosage administration.

Using NMR-based metabonomics it is possible to ‘bridge the gap’ between biofluid analysis and histopathology and to gain real insight into the mechanisms of drug efficacy at a molecular level. Thus, we applied NMR-based metabonomics to analyze in a holistic way for marker metabolites and to identify signals that can efficiently differentiate the groups, which can hardly be found by the conventional pharmacological methods.

In the present study, we explored the pattern recognition analysis such as PCA and OPLS-DA model to identify the metabolites that were characteristic of the group with hyperlipidemia and drug intervention. It was observed that higher concentrations of lipids (triglycerides and cholesterol), lactate, alanine together with lower concentrations of choline-containing compounds (e.g. phosphocholine, PtdCho, GPC), betaine in plasma and liver samples from hyperlipidemic hamsters compared to controls. However, the levels of these metabolites reversed when hyperlipidemic hamsters were treated with Simvastatin or WS070117. The intact tissues *ex vivo* assay by high resolution magic-angle spinning (HR MAS) NMR spectroscopy also showed that the pronounced differences in hepatic lipid content and choline-containing compounds between hyperlipidemic golden hamsters treated and untreated with WS070117. For the detection of lipid metabolites, NMR-based metabonomics presented consistent results with the biochemistry method. Moreover, this technology can find many other metabolites that significant contributed to distinguish among the groups.

More importantly, the results of NMR-based metabonomics gave us highlight on WS070117 that it had different hypolipidemic feature compared to Simvastatin. Here, Simvastatin mainly show a lipid-lowering effect and hardly made changes in low molecular weight metabolites that perform a vital role in lipid metabolism. Further study revealed that the main differential endogenous metabolites identified in liver and plasma of hyperlipidemic hamsters responded to WS070117 treatment were lipids, lipid-metabolism-related molecules, and some key amino acids. These observations summarized below increased our understanding of the metabolism pathways involved in the lipid lowing effect of WS070117 on HFD induced hyperlipidmia.

The changes of macromolecules in plasma caused by WS070117 contained apparent lower levels of LDL/VLDL, O-Ac and some of the lipid signals including CH_2_-CH_2_, CH = CH, C2 and C3 protons of fatty acids, accompanied with higher levels of HDL and PtdCho than those in hyperlipidemic hamsters. An increase in the signal at δ3.22 ppm (PtdCho) observed is consistent with the higher level of HDL since PtdCho is the most predominant lipid in the HDL fraction [19]–[21]. The total lipid concentration in the plasma of hyperlipidemic hamsters with administration of WS070117 was lower than that in untreated hyperlipidemic ones, as indicated by the loadings of resonances at δ2.26 ppm and δ1.58 ppm, contributed from the C2 and C3 protons of fatty acids. The micro-molecules in plasma of hyperlipidemic hamsters treated by WS070117 changed, including a reduction in the signals of lactate and alanine accompanied with an increase in betaine and GPC. These micro-molecular is involved in various pathway of energy metabolism. It was found lower lactate and total lipid levels in hyperlipidemic hamsters administrated with WS070117, suggesting that the rate of glycolysis was reduced and the energy consumption was switched to lipid oxidation [22]. Much of the alanine released from skeletal muscle comes from transamination of pyruvate formed during glycolysis and it can be transported in the blood to provide a liver gluconeogenesis substrate through the glucose-alanine cycle [23]. Therefore, the reduction in alanine caused by WS070117 may indicate that muscle proteins degration and liver gluconeogenesis was inhibited.

In liver tissues from hyperlipidemic hamsters, WS070117 treatement decreased levels of lipids (e.g.CH_3_, (CH_2_)_n_, CH_2_-CH = , CH_2_CO etc.), lactate, alanine and increased levels of choline-containing compounds (e.g. phosphorcholine, GPC), and betaine. Triglycerides are normally exported from the liver in the form of VLDL particles, which also contain relatively high concentrations of free cholesterol. Since VLDL synthesis requires the availability of phospholipids, particularly PtdCho, an insufficiency of PtdCho or its precursors can lead to decreased secretion of triglycerides from the liver and hence fatty liver. WS070117 increased levels of phospholipids (i.e. PtdCho) in hyperlipidemic hamsters, so it can promote triglycerides export from the liver to prevent fat accumulation. WS070117 was also found to increase hepatic GPC, which may be an indicator of liver “functionality” [13]. It was reported that betaine decreased the contents of hepatic cholesterol and total lipids in rats consuming a high-cholesterol diet [24].

In summary, these findings showed that NMR-based metabolomics did give a better picture of the multiparametric response to hyperlipidemia and pharmacological intervention, which confirmed by the significant deviation of biomarkers in the model group compared with normal controls and their recovery in treatment groups. Most interesting, the metabonomic study provided a visualized pattern and relative quantitative estimation to evaluate the effect of WS070117. In previous studies, WS070117 proved to be an activation agent of AMPK while simvastatin is a classical HMG-CoA reductase inhibitor. The different targets may result in the metabonomic diversity of WS070117 and simvastatin. Furthermore, future investigations of the relevance between metabolic profiles and target activation are needed to generate a more complete understanding of the hypolipidemic effects of WS070117.

## Materials and Methods

### Ethics statement

All protocols in this study were approved by the Medical Ethic Committee of Peking Union Medical College and were in accordance with National Institutes of Health regulations for the care and use of animals in research.

### Diets

The normal standard chow diet was purchased from Beijing HFK Bioscience Co. Ltd. The HFD was prepared by mixing pellets from the normal diet with lard oil (200 g/kg HFD) and cholesterol (2 g/kg HFD).

### Care and maintenance of animals

Twelve-month-old Syrian golden hamsters were purchased from Vital River Laboratory Animal Technology Co. Ltd. Twenty adult male Syrian golden hamsters (90–110 g) were acclimatized for 7 days in cages prior to model construction. They were given ad libitum access to food and water and maintained on a 12 h light/dark cycle (21±2°C with a relative humidity of 45±10%).

### Hyperlipidemic model construction

The animals were then randomly allocated into two groups, control (n = 5) and hyperlipidemic (n = 15). The hamsters that served as control group were fed ad libitum access to the standard chow, whereas the hyperlipidemic hamsters were fed a high-fat diet (Institute of Laboratory Animal Sciences, Beijing) 12g/animal/day quantitative feed to prevent animal store too much food in their cheek pouch for 2 weeks to establish the hyperlipidemic model.

### Drug administration

The hyperlipidemic hamsters were divided into three groups based on triglyceride and cholesterol levels in serum and body weight; these were the model group and the simvastatin- or WS070117-treated groups (2 mg·kg^−1^) (n = 5). The control and model hamsters were orally administered 2.5% carboxymethyl cellulose sodium (CMC-Na). All drugs were given once daily by oral gavage for 10 weeks. The animals in control and model group received the same volume of CMC-Na as that of the drug-treated group. The high fat diet was also administered the same quantity as model construction during the drug intervention.

### Ultrasonographic imaging

On the 10th week of drug administration and following an overnight fasting, the animals were anesthetized by inhalation of 1.5%–2% isoflurane gas. After removing hair from the abdomen and anchoring the limbs to a constant temperature circuit platform in a supine position, the animals were examined for their livers using an ultra imager (VisualSonics Vevo 770® High-Resolution Imaging System) equipped with a real-time micro-visualization scan-head probe.

### Measurement of lipid contents in serum

Hamsters were anesthetized with an intra-peritoneal injection of 3% sodium pentobarbital (2 ml per kg). An abdominal incision was made to expose the liver and inferior vena cava. Blood (3–4 ml) was withdrawn from the abdominal aorta and collected in tubes with or without heparin for collecting plasma and serum, respectively. The contents of serum TG, TC, LDL-C, and HDL-C were measured using commercial enzymatic assays (BioSino Bio-technology and Science Inc). Each sample was assayed in duplicate.

### Measurement of the hepatic TC and TG contents

After blood collection, the liver was excised and weighed. The right lateral liver was frozen for biochemical analysis. One hundred milligrams of frozen liver tissue was thawed and homogenized in 2 ml of chloroform-methanol (2∶1). After homogenization, lipids were extracted by rocking the samples for 1 h at room temperature followed by centrifugation at 5,000 g for 10 min. One milliliter of lipid extract was dried under a nitrogen stream and re-dissolved in 1 ml of isopropanol. TC and TG contents were measured using commercial kits.

### Oil Red O staining

Sections of liver tissue were fixed in 4% paraformaldehyde and dehydrated in a 30% sucrose solution at room temperature. Tissues were then immersed in Optimal Cutting Temperature (OCT) solution on dry ice. Three hamsters were randomly picked from each group and 3 different tissue sections from each liver were processed and stained using routine laboratory procedures.

### Sample collection for NMR analysis

#### Plasma and intact liver collection

Blood samples in heparinized tubes were centrifuged at 12,000 g for 15 min and the supernatant was collected. The left lateral lobe was taken from the total liver and immediately snap-frozen in liquid nitrogen. Both plasma and liver samples were stored at 193 K for ^1^H-NMR analysis.

#### Liver extraction procedure

Samples of intact frozen livers (∼0.5 g) were weighed and transferred into glass vials. The samples were homogenized with 2 ml of methanol and 425 µl water. One ml chloroform was added to the sample and vortexed. Then additional 1 ml chloroform and 1 ml water were added and the samples were vortexed again. The samples were kept on ice for 15 min and centrifuged at 1,000 g for 15 min at 277 K. The solution was separated into an upper methanol/water phase (with aqueous metabolites) and a lower chloroform phase (with lipophilic compounds), separated by protein and cellular debris. The upper and lower layers were transferred into separate glass vials. The solvent was removed under a stream of nitrogen. Aqueous and lipophilic extracts were stored at 193 K until analysis [25].

### ^1^H-NMR spectroscopy

All NMR experiments were performed on a Bruker AVANCE III-500 spectrometer (Bruker Biospin, Germany) operating at a ^1^H frequency of 500.13 MHz. Plasma spectra were recorded at 298 K by a triple resonance inverse TXI [^1^H, ^13^C, ^15^N]-xyz triple axis gradient probe, and data for intact liver samples were acquired at 277 K using HR MAS probe at a spinning rate of 4 kHz. A 90°pulse length was adjusted for each sample. 128 transients were collected into 32 k data points for each spectrum with a spectral width of 20 ppm and a recycle delay of 4.0 s.

Three kinds of ^1^H NMR spectra were acquired: a standard one-dimensional pulse sequence using the first increment of the NOESY pulse sequence to achieve water pre-saturation, a Carr-Purcell-Meiboom-Gill (CPMG) pulse sequence [25] to enhance the contribution of low molecular weight metabolites, and a diffusion-edited experiment using a bipolar pulse pair-longitudinal eddy current delay (BPP-LED) pulse sequence [25]–[27] to observe the lipid contents of lipoproteins in plasma. For the standard one-dimensional experiment, the mixing time (t_m_) was 100 ms. For the CPMG experiment, a spin-spin relaxation delay of 320 ms was used for each sample, and water signal irradiation was applied during the recycle delay. For the BPP-LED experiment, a sine shaped gradient with strength of 32 G/cm and duration of 2.5 ms was followed by a delay of 400 µs to allow for the decay of eddy currents. A diffusion delay of 120 ms and a delay T_e_ of 5 ms were used. A line-broadening factor of 0.3–1 Hz was applied to FIDs before Fourier transformation.

### ^1^H-NMR spectroscopy of plasma

Thirty µl of plasma was added to 60 µl of 0.9% saline (D_2_O∶H_2_O = 1∶9) containing 0.1% sodium 3-trimethylsilyl-propionate-2,2,3,3,-d4 (TSP) (an internal standard, chemical shift δ 0.0 ppm) in Eppendorf tubes. Samples were centrifuged at 12,000×g for 5 min at 298 K, and 60 µl of sample was transferred into 1.7-mm NMR tubes. 1D NOESY, CPMG and BPP-LED spectra were recorded.

### HR-MAS ^1^H-NMR spectroscopy of intact liver

All material in contact with the tissue was cooled to at least 277 K to reduce degradation during the sample preparation process. Frozen samples taken from a 193 K freezer were placed in a cryo-vial in liquid N_2_ until insertion into a 4-mm (o.d.) ZrO_2_ rotor. The pre-cooled rotor was filled with cooled D_2_O with 0.1% TSP after sample insertion. Spherical inserts were used to limit the rotor inner volume to 12 µl. ^1^H-NMR spectra of liver tissues were recorded using a standard 1D-NOESY and the CPMG pulse sequence.

### ^1^H-NMR spectroscopy of liver extracts

The aqueous extracts were reconstituted in 80 µl D_2_O containing 0.1% TSP. After centrifugation (1,000 g, 5 min), 60 µl of the supernatant were transferred to 1.7-mm NMR tubes. The ^1^H-NMR spectra of aqueous extracts were recorded using a water suppression pulse sequence, such as 1D NOESY-presat. For recording the ^1^H-NMR spectra of the lipophilic extracts, pre-saturation of the water resonance is not required, and thus a simple 90° pulse-acquire sequence was used.

### Relative quantification and statistical analysis

Relative intensities of some specific metabolites were determined using a selective signal of each metabolite from normalized spectra and results were represented as mean ± SD.

The other biochemical data were also expressed as the mean ± SD of five hamsters per group. Statistical analysis was performed using one-way ANOVA. A calculated P-value of less than 0.05 was considered statistically significant.

### Metabolite identification

Both visual inspection of the raw spectral data and statistical analysis were applied to identify discriminating peaks. Plasma and liver metabolites were identified with reference to published literature [28]–[29] and the Chenomx NMR Suite (Chenomx, Calgary, Canada).

### Data reduction of NMR spectra and pattern recognition (PR) analysis

The acquired NMR spectra were referenced to the chemical shift of TSP. Following phase and baseline correction, the ^1^H-NMR spectra were automatically reduced to ASCII files using AMIX software (Analysis of MIXtures software v. 3.0, Bruker Biospin). Each spectrum over the range of 0.5–6.0 ppm was data reduced and normalized to the total of all the resonance integral regions. The regions containing the resonance from residual water (4.7–5.1 ppm) were excluded. Residual ethanol resonances (1.17–1.23 ppm and 3.62–3.67 ppm) were also removed. Each reduced “buckets' has an equal width of 0.04 ppm and each integral region was thus effectively standardized to a ratio of the total metabolites detected in the sample. The ASCII files were imported into Microsoft Excel for labeling and then imported into SIMCA-P12.0 (Umetrics, Umeå, Sweden) for the PR analysis. Prior to analyses, the values of all variables were centered and scaled.

Principal components analysis (PCA) was used to reduce the dimensionality of the data sets and to produce an overview of the data set. PCA was also applied to identify outliers and detect data grouping or separation trends. The corresponding loadings plots were used to identify which spectral variables contribute to the positioning of the samples on the scores plot and hence the variables that influence any observed separation in the dataset.

Data were visualized with the PC scores plots, where each point represents an individual spectrum of a sample, and loadings plots, where each point represents a single NMR spectral region or chemical shifts. From the score and loading plots, classification of samples and the biochemical components responsible for the classification, respectively, can be shown.

The supervised pattern recognition (PLS-DA) was more focused on the actual class discriminating variation in the data compared to the unsupervised approach (PCA). PLS-DA is a frequently used PLS-based classification method in which the response variables are categorical (dummy variables describing the categories) and describe the class membership of the statistical units. PLS-DA aims to find the best possible discriminant function (model) separating classes of observations based on their X variables. When group separation was not satisfied based on PLS-DA, the data were further processed using orthogonal signal correction (OSC) which removes linear combinations of variables X orthogonal to the Y vector of the dependent variables, and thus eliminates inter-subject variability and generates maximum class separations [30]. The PLS-DA model was validated by permutation method with describing R^2^Y and Q^2^Y values. The parameter Q^2^ was used to provide an estimation of the predictive capability of the PLS-DA models with Q^2^>0.5 considered ‘good’ and Q^2^>0.9 ‘excellent’. The parameter R^2^ describes the explained variation and how well the data can be mathematically reproduced by the training model. Commonly, R^2^Y provides an estimate of how well the model fits the Y data, whereas Q^2^Y is an estimate of how well the model predicts the Y. The fact that both Q^2^Y and R^2^Y are close to 1 indicates an excellent model, whereas the poor ratio of them is likely to be the onset of model overfitting [31].

## Supporting Information

Table S1 ^1^H Chemical shift assignment of the metabolites in hamster plasma and liver samples.
(DOC)
