# Supplementary material for: Beneficial Metabolic Effects of 2′,3′,5′-tri-acetyl-N6- (3-Hydroxylaniline) Adenosine in the Liver and Plasma of Hyperlipidemic Hamsters
Source: PLoS One. 2012 Mar 28;7(3):e32115. doi: 10.1371/journal.pone.0032115 (PMC3314636; doi:10.1371/journal.pone.0032115)
Supplement: Table S1 — 1H Chemical shift assignment of the metabolites in hamster plasma and liver samples. (DOC) [file pone.0032115.s001.doc]

**Table S1. ^1^H Chemical shift assignment of the metabolites in plasma, and liver from hamsters**

| **Biological matrices** | **Metabolites** | **Moieties** | **δ^1^H(ppm)and multiplicity** | |
| --- | --- | --- | --- | --- |
| **Plasma** | Leucine | γCH_3_ | 0.95(d) |  |
|  | Valine | γCH_3_,γ'CH_3_ | 0.98(d),1.03(d) |  |
|  | Isoleucine | δCH_3_ | 1.01(d) |  |
| **Intact Liver&Liver**  **Aqueous Extracts** | 3-HB  Lactate  Lysine  Alanine  Acetate  N-Acetyl glycoproteins  O-Acetyl glycoproteins  Methionine  Acetoacetate  Pyruvate  Glutamine  Creatine  Betaine  Taurine  TMAO  Tyrosine  Choline  Phosphocholine/GPC  Phosphatidylcholine  α-Glucose  β-Glucose  Glucose/amino acids resonances  Lipids(VLDL/LDL)  Lipids(VLDL/LDL)  Lipids(HDL)  Lipids(HDL)  Lipids  Lipids  Lipids  Lipids  Unsaturated lipids  Leucine  Valine  Isoleucine  3-HB  Lactate | γCH_3_  βCH_3_,αCH  γCH_2_  βCH_3_  βCH_3_  CH_3_  CH_3_  S-CH_3_  CH_3_  CH_3_  γCH_2_  N-CH_3_  N(CH_3_)_3_  N-CH_2_,S-CH_2_  N(CH_3_)_3_  CH,CH  N(CH_3_)_3_  N(CH_3_)_3_  N(CH_3_)_3_  CH  CH  ring protons/α-CH  -CH_3_  -(CH_2_)_n_-  -CH_3_  -(CH_2_)_n_-  -CH_2_CH_2_CO  -CH_2_CH=  -CH_2_CO  =CHCH_2_CH=  -CH=CH-  γCH_3_  γCH_3_,γ'CH_3_  δCH_3_  γCH_3_  βCH_3_,αCH | 1.20(d)  1.34(d),4.11(q)  1.45(m)  1.46(d)  1.94(s)  2.04(s)  2.06(s)  2.13(s)  2.29(s)  2.41(s)  2.14(),2.46(m)  3.03(s)  3.26(s)  3.24(t),3.42(t)  3.26(s)  6.87(m),7.16(m)  3.20(s)  3.22(s)  3.22(s)  5.22(d)  4.62(d)  3.4-4.0  0.9(t)  1.30(m)  0.86(t)  1.26(m)  1.61(m)  2.02(m)  2.26(m)  2.78(m)  5.34(m)  0.95(d)  0.98(d),1.03(d)  1.01(d)  1.20(d)  1.34(d),4.11(q) |  |

**Table S1. Continued.**

| **Biological matrices** | **Metabolites** | **Moieties** | **δ^1^H(ppm)and multiplicity** | |
| --- | --- | --- | --- | --- |
| **Intact Liver&Liver**  **Aqueous Extracts**  **Liver Aqueous Extracts** | Lysine  Alanine  Acetate  Glutamine  Acetoacetate  Betaine  Taurine  TMAO  Choline  Phosphocholine/GPC  α-Glucose  β-Glucose  glucose/glycogen/α-H  amino acids  Glycogen  Lipids  Lipids  Lipids  Lipids  Lipids  Lipids  Unsaturated lipids  Bile acid  Glutamate  Glutathione  Succinate  Aspartate  Tyrosine  UDP-Glucose  NAD^+^  NADP^+^  Inosine  AMP  Fumarate  Histidine  Nicotinurate  Adenine  Adenosine | γCH_2_  βCH_3_  βCH_3_  γCH_2_  CH_3_  N(CH_3_)_3_  N-CH_2_,S-CH_2_  N(CH_3_)_3_  N(CH_3_)_3_  N(CH_3_)_3_  CH  CH  ring protons/α-CH  CH  -CH_3_  -(CH_2_)_n_-  -CH_2_CH_2_CO  -CH_2_CH=  -CH_2_CO  =CHCH_2_CH=  -CH=CH-  C_18_H_3_  βCH_2_, γCH_2_  βCH_2_, γCH_2_, S-CH_2_  CH_2_  βCH_2_, αCH  CH,CH  ring protons  ring protons  ring protons  ring protons  ring protons  CH=  ring protons  ring protons  ring protons  ring protons | 1.45(m)  1.46(d)  1.94(s)  2.14(),2.46(m)  2.29(s)  3.26(s)  3.26(t),3.42(t)  3.26(s)  3.20(s)  3.22(s)  5.22(d)  4.62(d)  3.4-4.0  5.38-5.44  0.9-0.94(m)  1.30-1.34(m)  1.61(m)  2.02(m)  2.26(m)  2.78(m)  5.30(m)  0.76(m )  2.06(m), 2.35(m)  2.16(m),2.54(m),2.96(dd)  2.39(s)  2.66(dd),2.80(dd),3.89(dd)  6.87(m),7.16(m)  6.00(m),7.94(d)  6.03(d),6.09(d),8.14 (s)  8.84(d),9.14(d),9.33(s)  6.09(d),8.19(s),8.82(d)  9.16(d),9.33(s)  6.06(d),8.19(s),8.31(s)  6.12(d),8.23(s),8.58(s)  6.52(s)  7.07(s),7.90(s)  8.27(t),8.97(m)  8.16(s)  6.03(d),8.12(s),8.28(s) |  |

**Table S1. Continued.**

| **Biological matrices** | **Metabolites** | **Moieties** | **δ^1^H(ppm)and multiplicity** | |
| --- | --- | --- | --- | --- |
| **Liver Lipophilic**  **Extracts** | Cholesterol  Triglycerides  Phospholipids    Fatty acid residues | C_18_**H**_3_  C_26_**H**_3_,C_27_**H**_3_,C_21_**H**_3_  C_19_**H**_3_  C_3_**H**  C_6_**H**  C_1_**H** and C_3_**H** of glycerol  C_1_**H** and C_3_**H** of glycerol  C_2_**H** of glycerol  N^+^(C**H**_3_)_3_ of PC and SM  C**H**_2_-NH_2_ of PE  ω-C**H**_3_  ω-C**H**_3_ of total omega-3  fatty acid  (C**H**_2_)_n_  COCH_2_-C**H**_2_  β-C**H**_2_ of ARA+EPA  -C**H**_2_-CH=  -CO-C**H**_2_  α and β C**H**_2_ of DHA  -CH=CH-C**H**_2_-CH=CH-  of linoleic acid  (CH=CH-C**H**_2_-CH=CH)_n_  n>1  C**H**=C**H** | 0.67(s)  0.87-0.92(d)  1.01(s)  3.50  5.37  4.15(dd)  4.29(dd)  5.25(q)  3.20(s)  3.60(m)  0.88(t)  0.98(t)  1.30(m)  1.59(m)  1.67(m)  2.04(m)  2.30(m)  2.38(m)  2.75(t)  2.80(m)  5.36(m) |  |

s=singlet; d=doublet; dd=double doublet; t=triplet; q=quartet; m=multiplet.
